# Differential role of MyD88 and TRIF signaling in myeloid cells in the pathogenesis of autoimmune diabetes

**DOI:** 10.1371/journal.pone.0194048

**Published:** 2018-03-09

**Authors:** Ariadne Androulidaki, Laurens Wachsmuth, Apostolos Polykratis, Manolis Pasparakis

**Affiliations:** 1 Institute for Genetics, University of Cologne, Cologne, Germany; 2 Cologne Excellence Cluster on Cellular Stress Responses in Aging-Associated Diseases (CECAD), University of Cologne, Cologne, Germany; 3 Center for Molecular Medicine (CMMC), University of Cologne, Cologne, Germany; Children's Hospital Boston, UNITED STATES

## Abstract

Type 1 diabetes (T1D) is caused by the autoimmune destruction of the insulin-producing pancreatic beta cells. While the role of adaptive immunity has been extensively studied, the role of innate immune responses and particularly of Toll- like Receptor (TLR) signaling in T1D remains poorly understood. Here we show that myeloid cell-specific MyD88 deficiency considerably protected mice from the development of streptozotocin (STZ)-induced diabetes. The protective effect of MyD88 deficiency correlated with increased expression of the immunoregulatory enzyme indoleamine 2,3-dioxygenase (IDO) in pancreatic lymph nodes from STZ-treated mice and in bone marrow-derived dendritic cells (BMDC) stimulated with apoptotic cells. Mice with myeloid cell specific TIR-domain-containing adapter-inducing interferon-β (TRIF) knockout showed a trend towards accelerated onset of STZ-induced diabetes, while TRIF deficiency resulted in reduced IDO expression *in vivo* and *in vitro*. Moreover, myeloid cell specific MyD88 deficiency delayed the onset of diabetes in Non-Obese Diabetic (NOD) mice, whereas TRIF deficiency had no effect. Taken together, these results identify MyD88 signaling in myeloid cells as a critical pathogenic factor in autoimmune diabetes, which is antagonized by TRIF-dependent responses. This differential function of MyD88 and TRIF depends at least in part on their opposite effects in regulating IDO expression in phagocytes exposed to apoptotic cells.

## Introduction

Type 1 diabetes (T1D) is an autoimmune disease caused by the immune cell-mediated destruction of pancreatic β-cells. A broad immune deregulation is believed to contribute to the pathogenesis of T1D [1]. Studies in animal models of T1D have made fundamental contributions to the understanding of the mechanisms triggering autoimmune destruction of β-islets. The nonobese diabetic (NOD) mice constitute a valuable genetic model of T1D mimicking the human disease [2, 3]. An experimental model of T1D can also be induced by the repetitive administration of low-dose STZ, which causes β-cell death and triggers autoimmune destruction of pancreatic islets [4, 5]. Studies in these models showed that complete β-cell destruction is preceded by insulitis, characterized by invasion of pancreatic islets by a mixed population of lymphocytes and antigen presenting cells (APCs) including macrophages and dendritic cells (DCs).

Earlier studies highlighted the key role of T lymphocytes in the mechanisms leading to islet destruction and diabetes [1], whereas the role of the innate immune system in diabetes development became more evident only in recent years [6]. A physiological wave of β-cell apoptosis in the pancreas is believed to be the crucial initiating event in T1D [7]. Recruitment of phagocytes precedes T-cell infiltration and is believed to be triggered by the accumulation of apoptotic β-cells [8, 9]. The interaction of phagocytes with apoptotic or secondary necrotic β-cells has been suggested to induce the initial response by stimulating the priming of diabetogenic T- cells in a TLR2-dependent manner, as genetic deletion of TLR2, but not TLR4, significantly reduced diabetes incidence in NOD mice, as well as in mice treated with STZ [10].

The adapter MyD88 is essential for signaling by all TLRs except TLR3 that uses the adapter TRIF instead, while TLR4 utilizes both the MyD88 and TRIF pathways [11]. Various studies suggested an important role for MyD88 in the induction of autoimmunity [12–15]. In contrast to MyD88, the role of the TRIF-mediated branch of TLR signaling in autoimmunity is less studied, with existing evidence suggesting that TRIF-mediated signaling protects against autoimmunity [16, 17]. Specifically for T1D, MyD88 deficiency completely prevented diabetes development in NOD mice in a microbiota-dependent manner [18]. In contrast, TRIF signaling was suggested to mediate a microbiota-dependent protective effect in the development of T1D in NOD mice [19]. Another study suggested that MyD88 deficiency worsens STZ- induced injury and T1D development by regulating the size of pancreatic islets and their response to injury [20]. Furthermore, it has been shown that TRIF deficient mice have increased β-cell mass and suffer from hyperglycemia associated with β-cell dysfunction [21]. Due to the important role of both MyD88 and TRIF in pancreatic β-cells, the results obtained from studies addressing the role of these molecules in T1D development using conventional knockout mice are difficult to interpret. Therefore, studies using cell-specific knockouts are required to address the role of MyD88 and TRIF signaling in the immunological mechanisms triggering islet autoimmunity and T1D. Here we employed conditional gene targeting in mice to address the myeloid cell-specific role of MyD88 and TRIF in the development of T1D using the NOD and the STZ-induced models.

## Materials and methods

### Mice

MyD88gfpFL and TRIFmcFL mice were generated as described before [22, 23] and were maintained in C57Bl/6N genetic background. CD11cCre [24], LysMCre [25] and Cre-Deleter [26] mice were backcrossed for more than 10 generations into the C57Bl/6N genetic background. *Myd88*^-/-^ and *Trif*^-/-^ mice were generated by crossing mice carrying the respective floxed lines with Cre-Deleter transgenics. Mice were maintained at the SPF animal facility of the Institute for Genetics, University of Cologne, kept under a 12 h light cycle, and given a regular chow diet *ad libitum*. Mice were sacrificed with cervical dislocation. All animal procedures were conducted in accordance with European, national and institutional guidelines and protocols and were approved by the responsible government authority: Landesamt für Natur, Umwelt und Verbraucherschutz NRW. (Animal Licence: LANUV NRW 84–02.04.2013.A266).

### Streptozotocin- induced diabetes induction

Mice in C57Bl/6N genetic background were used for STZ-induced diabetes. Female 8–12 week old mice were given five consecutive daily intraperitoneal injections of 50mg/kg body weight STZ (Sigma) diluted in 10mM citrate buffer in order to induce diabetes. Control mice were injected with buffer alone. Blood glucose levels were examined seven days after the final STZ injection, and every week from that time on by obtaining blood from the lateral saphenous vein and measuring glucose concentration with a glucometer (Accu-Check instant, Boehringer Mannheim Corporation, Indianapolis, IN, USA). Mice with blood glucose levels greater than 300 mg/dl for two consecutive measurements were considered diabetic.

### Preparation of bone marrow derived dendritic cells and cell culture

Bone marrow- derived dendritic cells (BMDC) were prepared according to standard protocol. Briefly, mice were sacrificed and tibiae were removed. Marrow from tibiae was isolated and erythrocytes were lysed. Cells were washed and resuspended in DC medium (RPMI 16–40, 10%FBS, 1% Pen/strep, 50uM 2-ME, L-glutamine 2mM) plus 20ng/ml rmGM-CSF (Peprotech) at a concentration of 2x10^6^ cells per 100mm dish. After 10 days, cells were replated at 1x10^6^/ml for experiments in DC medium without GM-CSF. For the relevant experiments cells were stimulated with 10ng/ml LPS (Alexis Biochemicals) or with freshly prepared primary apoptotic cells at a ratio 1:1.

### Isolation of peritoneal cells

Mice were sacrificed and the peritoneal cavity was washed with sterile PBS supplemented with 1% BSA. Cell suspension was filtered (70μm), washed twice, cells were counted and used for experiments.

### Preparation of apoptotic cells

For the preparation of primary apoptotic splenocytes or thymocytes, wild type mice were sacrificed, spleens or thymuses were removed respectively, gently dissociated and erythrocytes were lysed. Splenocytes were placed in medium with 1% FCS and stimulated with 1mM staurosporine (Sigma) for 12hrs. On the next day, splenocytes were washed, counted and used for stimulation experiments. Thymocytes were placed in medium with 1% FCS and irradiated (20Gy). Thymocytes were left for 6 hours in the incubator allowing apoptotic changes to occur and then directly used for stimulation experiments. The degree of apoptosis at the time of stimulation in each preparation was checked by FACS analysis and was found to be 70–85% AnnexinV^+^ and 10–30% 7AAD^+^.

### Isolation of pancreatic islets

Islet isolation was performed according to published protocols [27]. Briefly, mice were sacrificed and perfused with collagenase. The pancreas was removed and digested. Islets were hand-picked, placed in 96-well plate in RPMI +1% FCS (20 islets per well) and stimulated for 24hrs with STZ (Sigma, 10mmol/L).

### qRT-PCR

Total RNA isolated from tissue (PLNs) or DCs in culture was reverse-transcribed to cDNA using a cDNA synthesis kit (Invitrogen). Quantitative real time-PCR (qRT-PCR) was performed using the Applied Biosystems 7900HT Fast Real-Time PCR System with TaqMan probes. TaqMan Gene Expression Assays (Applied Biosystems) were performed according to the manufacturers protocols. Each sample was measured in triplicate. Relative gene expression was normalized against *Gapdh*.

### Western blot analysis

BMD-DCs were plated at a concentration of 10^6^cells/ml. After stimulation cells were lysed in lysis buffer (150 mM NaCl, 1% NP-40, 0.1% SDS in a 50 mM Tris buffer at pH 7.5) including the Protease inhibitor tablets (cOmplete, Roche) and phosphatase inhibitors (PhosSTOP Roche). Proteins were size-separated on a denaturing 10% polyacrylamid gel using gel electrophoresis. After gel-electrophoresis proteins were transferred onto a PVDF membrane (Millipore) using the following protocol for semi-dry blotting. Primary and secondary antibodies used for detection of specific proteins. Immunodetection was done using ECL reagent from GE Healthcare. The following antibodies were used: RelB (sc-226 Santa Cruz, 1:1000), cRel (sc-71 Santa Cruz, 1:1000), HDAC1 (sc-7872 Santa Cruz, 1:1000), Tubulin-a (T6074 Sigma, 1:10000), Ido (Cat. #05–840 Millipore, 1:500), MyD88 (sc-11356 Santa Cruz, 1:1000), Actin (sc-1616 Santa Cruz, 1:1000), p100/ p52 (kindly provided from Pr. Dr. Gurisankar Ghosh)

### Histological analysis of tissue sections

Tissues were fixed overnight in 4% paraformaldehyde, embedded in paraffin and cut in 7-μm sections. To determine the severity of insulitis, 30–50 pancreatic islets from 5–6 parallel sections of different cut levels per mouse were stained with hematoxylin and eosin (H&E) according to standard protocols. The degree of insulitis was classified into four categories: 0, no insulitis; I, peri-insulitis with minimal lymphocytic infiltration in islets; II, invasive insulitis with islet destruction <50%, and III, severe invasive insulitis with islet destruction >50%. Sections were incubated with primary rabbit anti-CD3 (Abcam) and rat anti-F4/80 (Abd Serotec) antibodies, followed by secondary biotinylated anti-rabbit (Perkin Elmer) or anti-rat (Jackson ImmunoResearch Inc) antibodies. Stainings were visualized with the ABC Kit Vectastain Elite (Vector) and DAB substrate (DAKO).

### FACS analysis

For the flow cytometric analysis of the MyD88 and TRIF mice resident peritoneal cells were harvested from mice by lavage of the peritoneal cavity with PBS supplemented with 1% BSA. Single cell suspensions from spleens or pancreatic lymph nodes (PLNs) were prepared mechanically in 1% BSA in PBS and 70μm filtered. Erythrocytes were lysed, cells were washed and samples were directly stained according to standard protocols with the respective FACS antibodies. FACS analysis was performed at the FACSCalibur (BD). Data were analyzed using the FlowJo 7.6.5 software. For sorting of GFP positive cells samples were prepared as described for FACS analysis and sorted using a FACS-Sorter (Beckman Coulter).

### Phagocytosis assay

Apoptotic splenocytes were prepared as described above, labelled with the fluorogenic dye Calcein-AM (Molecular Probes) for 30 min and washed. Calcein-AM is non- fluorescent but once loaded into cells is cleaved by endogenous esterases to produce highly fluorescent (FITC) calcein. BMDCs were stimulated with the labelled apoptotic splenocytes (1:1) for 3 or 8 hours and phagocytosis efficiency was assessed by FACS analysis measuring percentage of CD11c^+^ FITC^+^ cells.

### Apoptotic cell administration *in vivo*

Apoptotic thymocytes were prepared as described above. Mice were injected (i.p.) with 10^7^ apoptotic thymocytes or medium only for 18 hours. Peritoneal cells were collected and gene expression was determined by qRT-PCR analysis.

### Statistical analysis

Results are represented as the mean +/- standard deviation (SD). Statistical significance between experimental groups was determined by an unpaired two-tailed Student's t-test. The Fisher’s exact test was used for analysis of the diabetes incidence and the histological scoring. (* p<0.05, ** p<0.01 and *** p<0.001)

## Results

### Generation and characterization of myeloid-specific MyD88 and TRIF knockout mice

In order to study the cell-specific role of TLR signaling we employed mice carrying loxP-flanked *Myd88* [22] and *Trif* [23] alleles. By crossing these mice to *LysM-Cre* and *CD11c-Cre* transgenics we obtained mice with deficiency of MyD88 or TRIF specifically in myeloid cells. To assess the efficiency and specificity of Cre recombination in these mice we took advantage of the fact that Cre-mediated excision of the floxed genomic fragments activates the expression of GFP and mCherry respectively from the recombined *Myd88* and *Trif* alleles. These experiments showed that both the *LysM-Cre* and *CD11c-Cre* lines mediated recombination in cells assigned to the macrophage and dendritic cell lineages. FACS analysis of *Myd88*^FL/FL^, *LysM-Cre*^tg/wt^ mice (hereafter referred to as MyD88^LysM-KO^) showed that MyD88 was efficiently deleted in resident peritoneal CD11b^+^, F4/80^+^ or CD11c^+^ cells, as well as splenic neutrophils (CD11b^+^Gr1^+^), but not in B or T cells (S1 Fig). In *Myd88*^FL/FL^, *CD11c-Cre*^tg/wt^ mice (hereafter referred to as MyD88^CD11c-KO^) although we found substantial Cre recombination in CD11b^+^ and F4/80^+^ resident peritoneal cells, only CD11c^+^ cells displayed more than 85% deletion. Neutrophils as well as B- and T-cells did not display considerable recombination in MyD88^CD11c-KO^ mice (S1 Fig).

To confirm that Cre-mediated recombination also resulted in loss of MyD88 protein expression in the relevant cell types, we analyzed protein extracts from sorted resident peritoneal macrophages (F4/80^high^CD11b^+^ cells) from MyD88^LysM-KO^ mice and from sorted resident peritoneal dendritic cells (CD11c^+^CD11b^+^) from MyD88^CD11c-KO^ mice. Consistent with the results of FACS analysis of GFP expression, immunoblot analysis revealed efficient ablation of MyD88 protein expression from these cells (S1 Fig). Furthermore, to functionally assess MyD88 deficiency we stimulated resident peritoneal macrophages from *Myd88*^FL^, MyD88^LysM-KO^ mice or germ-line MyD88 knockout mice (*Myd88*^-/-^) with LPS. As shown in S1 Fig, the expression of MyD88-dependent genes such as *Ccl2*, *Il6* and *Cox2* was suppressed in macrophages from MyD88^LysM-KO^ as well as *Myd88*^-/-^ mice, providing further evidence that MyD88 was efficiently deleted in MyD88^LysM-KO^ mice.

FACS analysis of *Trif*^FL/FL^, *LysM-Cre*^tg/wt^ (hereafter referred to as TRIF^LysM-KO^) revealed efficient recombination in resident peritoneal CD11b^+^, F4/80^+^ or CD11c^+^ cells, as well as splenic neutrophils (CD11b^+^Gr1^+^), similarly to the results observed in MyD88^LysM-KO^ mice (S2 Fig). However, analysis of *Trif*^FL/FL^, *CD11c-Cre*^tg/wt^ mice (hereafter referred to as TRIF^CD11c-KO^) showed that the deletion efficiency within the CD11c^+^ population was about 50% (S2 Fig), considerably lower than the 85% deletion observed in MyD88^CD11c-KO^ mice. Analysis of CD11c^+^ cells from germ-line TRIF knockout mice (*Trif*^-/-^) mice showed mCherry expression in 90–95% of the cells, suggesting that the expression of mCherry in 50% of the cells reflects low deletion efficiency and is not due to expression of TRIF in only a fraction of these cells.

### Myeloid cell-specific MyD88 deficiency delayed and decreased the incidence of STZ- induced diabetes

**Fig 1:**
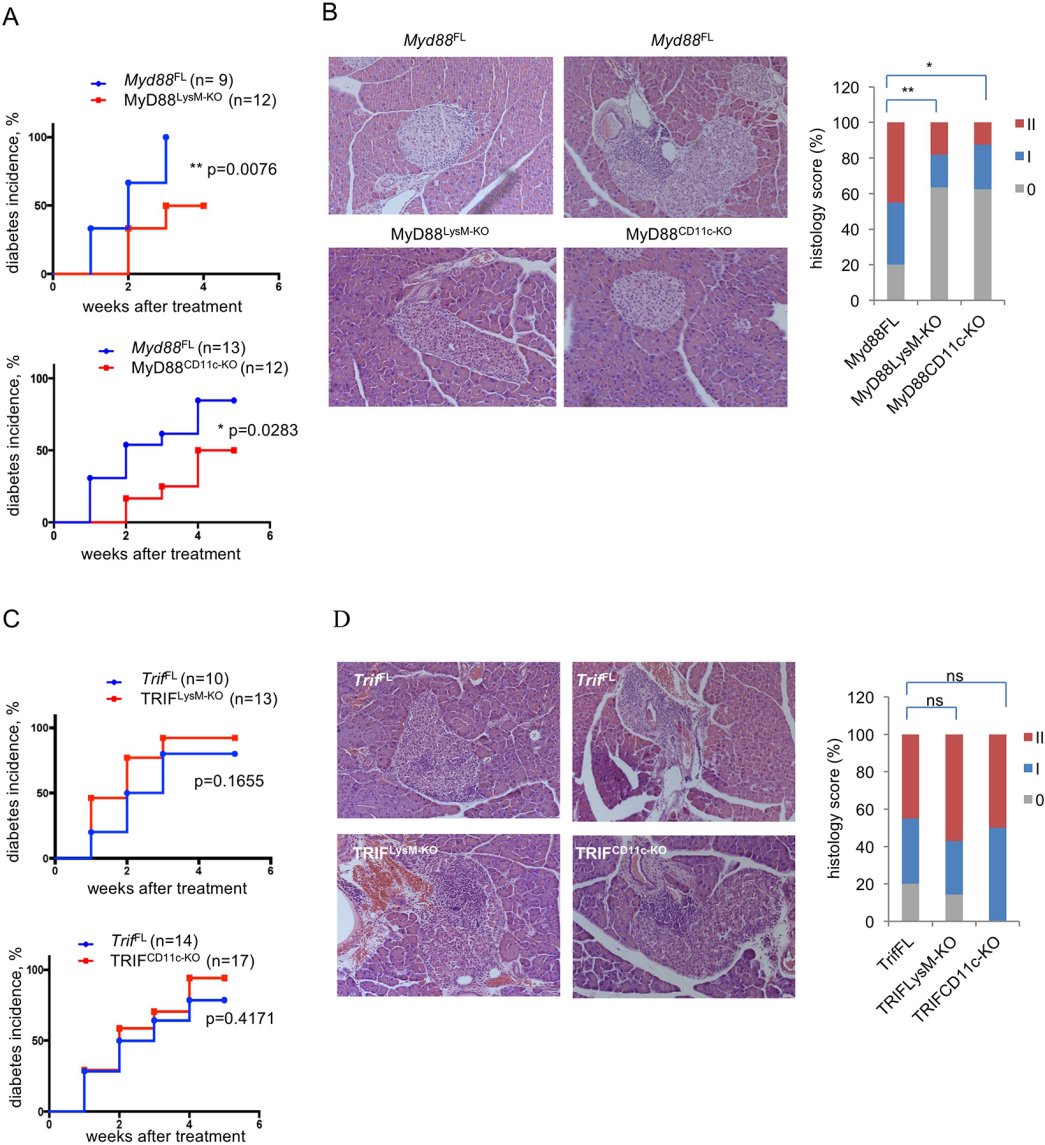
STZ-induced diabetes development in mice with myeloid cell-specific MyD88 or TRIF deficiency. (A and C) Graphs depicting the incidence of diabetes in mice with the indicated genotypes after treatment with 50 mg/kg STZ for five consecutive days. (B and D) Representative images and quantification of H&E stained paraffin sections of pancreatic tissue from mice with the indicated genotypes one week after completion of the STZ treatment. Graph depicts the percentage of mice with a given histology score per genotype: 0, no islet infiltrates, I, peri-insulitis, II, invasive insulitis. 30–50 islets per mouse were examined. MyD88^CD11c-KO^ (n = 8), MyD88^LysM-KO^ (n = 6), *Myd88*^FL^ (n = 9), TRIF^CD11c-KO^ (n = 6), TRIF^LysM-KO^ (n = 7) and *Trif*^FL^ (n = 11).

To assess the role of myeloid cell-specific MyD88 signaling in autoimmune diabetes, we used the STZ-induced model of T1D. Groups of 8–12 week old MyD88^LysM-KO^ or MyD88^CD11c-KO^ mice and their respective *Myd88*^FL/FL^ control animals (*Myd88*^*FL*^) were injected intraperitoneally with 50 mg/kg body weight STZ for five consecutive days. Control mice were injected with buffer alone. Blood glucose levels were examined weekly, starting seven days after the final STZ injection. Mice exhibiting blood glucose levels greater than 300 mg/dl in two consecutive measurements were considered diabetic. MyD88^LysM-KO^ mice showed delayed and decreased incidence of diabetes, with only 50% of the mice being diabetic 4 weeks after treatment, in contrast to their littermate control *Myd88*^FL^ mice that showed 100% diabetes incidence 3 weeks after treatment (Fig 1A). Furthermore, MyD88^CD11c-KO^ mice also showed considerably delayed and decreased diabetes incidence with only 50% of the mice developing diabetes compared to 90% of their littermate *Myd88*^FL^ mice (Fig 1A). STZ-induced injury is followed by immune cell infiltration in the pancreatic islets. In agreement with the delayed and reduced diabetes incidence, histological analysis of pancreatic sections revealed significantly reduced immune cell infiltrates in MyD88^LysM-KO^ and MyD88^CD11c-KO^ mice compared to their littermate controls one week after STZ administration (Fig 1B). Collectively, these results showed that MyD88 deficiency in myeloid cells considerably inhibited the development of diabetes in mice treated with STZ, suggesting that MyD88 signaling in myeloid cells promotes autoimmune responses leading to the development of T1D.

### Myeloid cell-specific TRIF deficiency did not considerably affect the development of STZ- induced diabetes

To assess the role of myeloid cell specific TRIF signaling in the development of STZ-induced diabetes we injected groups of 8–12 week old TRIF^LysM-KO^ or TRIF^CD11c-KO^ mice and their respective *Trif*^FL/FL^ littermates (*Trif*^FL^) with 50mg/kg body weight STZ for five consecutive days and monitored diabetes development by weekly measurement of blood glucose levels. TRIF^LysM-KO^ mice showed a trend towards accelerated diabetes development, however there were no statistically significant differences in disease onset or overall incidence with the control group (Fig 1C). The course and incidence of diabetes development in TRIF^CD11c-KO^ mice was not different from that of their littermate controls (Fig 1C). Consistent with these findings, histological analysis of pancreatic sections did not reveal significant differences in immune cell infiltration in TRIF^LysM-KO^ and TRIF^CD11c-KO^ mice compared to their littermate controls one week after STZ administration (Fig 1D). Therefore, TRIF deficiency in myeloid cells did not significantly alter diabetes incidence, although there was a trend towards accelerated onset of STZ-induced diabetes, indicating that TRIF signaling in myeloid cells may have a protective effect by delaying the development of autoimmune diabetes in response to islet injury.

### Differential expression of *Ido* and presence of Tregs in the PLNs of MyD88^CD11c-KO^ or TRIF^CD11c-KO^ mice after STZ

**Fig 2:**
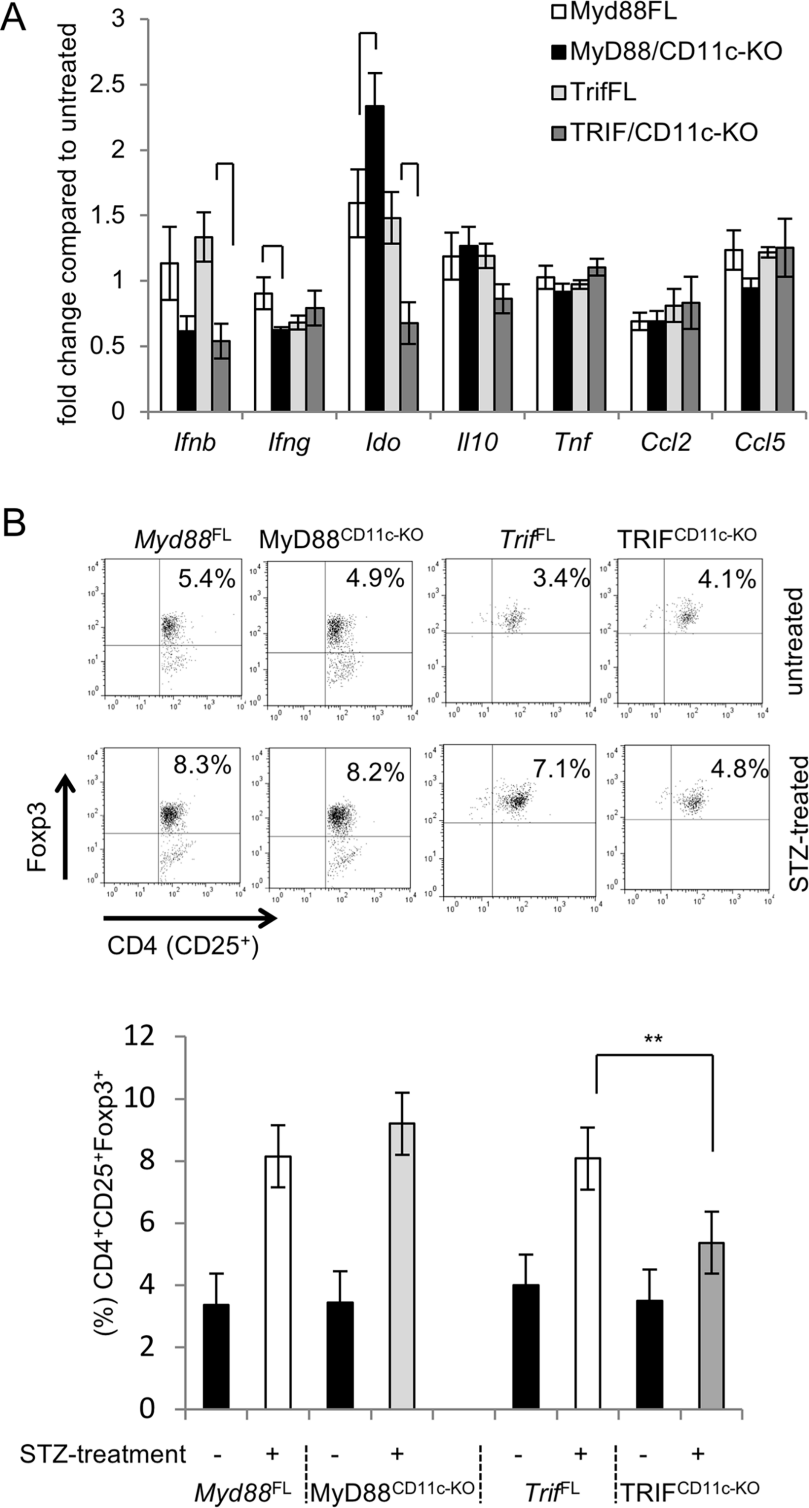
Differential effect of MyD88 and TRIF deficiency on *Ido* expression and Treg induction in PLNs of STZ-treated mice. (A) The mRNA expression of the indicated genes was measured by qRT-PCR in the PLNs of MyD88^CD11c-KO^ (n = 5), TRIF^CD11c-KO^ (n = 9) and their respective littermate control *Myd88*^FL^ (n = 4) and *Trif*^FL^ (n = 8) mice one week after the completion of STZ injections. Results are depicted as fold increase compared to untreated mice (buffer-only) of each genotype. (B) FACS analysis for Tregs (CD4^+^CD25^+^Foxp3^+^) in the PLNs of STZ-treated mice one week after the completion of the STZ injections. Representative plots of gated live, CD4^+^ PLN cells stained with CD25 and intracellular Foxp3. Bar graph shows quantification of CD4^+^CD25^+^Foxp3^+^ cells in STZ-treated *Myd88*^FL^ (n = 4), MyD88^CD111c-KO^ (n = 6), *Trif*^FL^ (n = 5) and TRIF^CD11c-KO^ (n = 6), as well as untreated *Myd88*^FL^ (n = 3), MyD88^CD111c-KO^ (n = 2), *Trif*^FL^ (n = 2) and TRIF^CD11c-KO^ (n = 2) mice.

Upon STZ-mediated islet injury dying beta cells are phagocytosed by antigen presenting cells (APCs), which then migrate from the pancreas to the PLNs and present (auto)antigens to naive T-cells [9]. Previous studies revealed that the predominant type of APCs transporting beta cell debris from the pancreas to the regional lymph nodes and initiating autoimmune responses are CD11b^+^CD8a^-^ DCs rather than macrophages [9]. Our results described above suggested that MyD88 and TRIF exhibit differential functions in myeloid cells, promoting or delaying respectively the development of STZ induced diabetes. To gain mechanistic insights on the roles of MyD88 and TRIF in the development of autoimmunity in this model, we focused on MyD88^CD11c-KO^ and TRIF^CD11c-KO^ mice. We first analyzed the expression of immunoregulatory molecules in PLNs from mice collected one week after the completion of STZ treatment. MyD88^CD11c-KO^ mice showed reduced expression of *Ifng*, but elevated expression of *Ido* (indoleamine 2,3-dioxygenase) (Fig 2A), an enzyme with potent immunosuppressive properties in DCs. Interestingly, the expression of *Ido* was considerably reduced in PLNs of TRIF^CD11c-KO^ mice compared to their littermate controls (Fig 2A).

**Fig 3:**
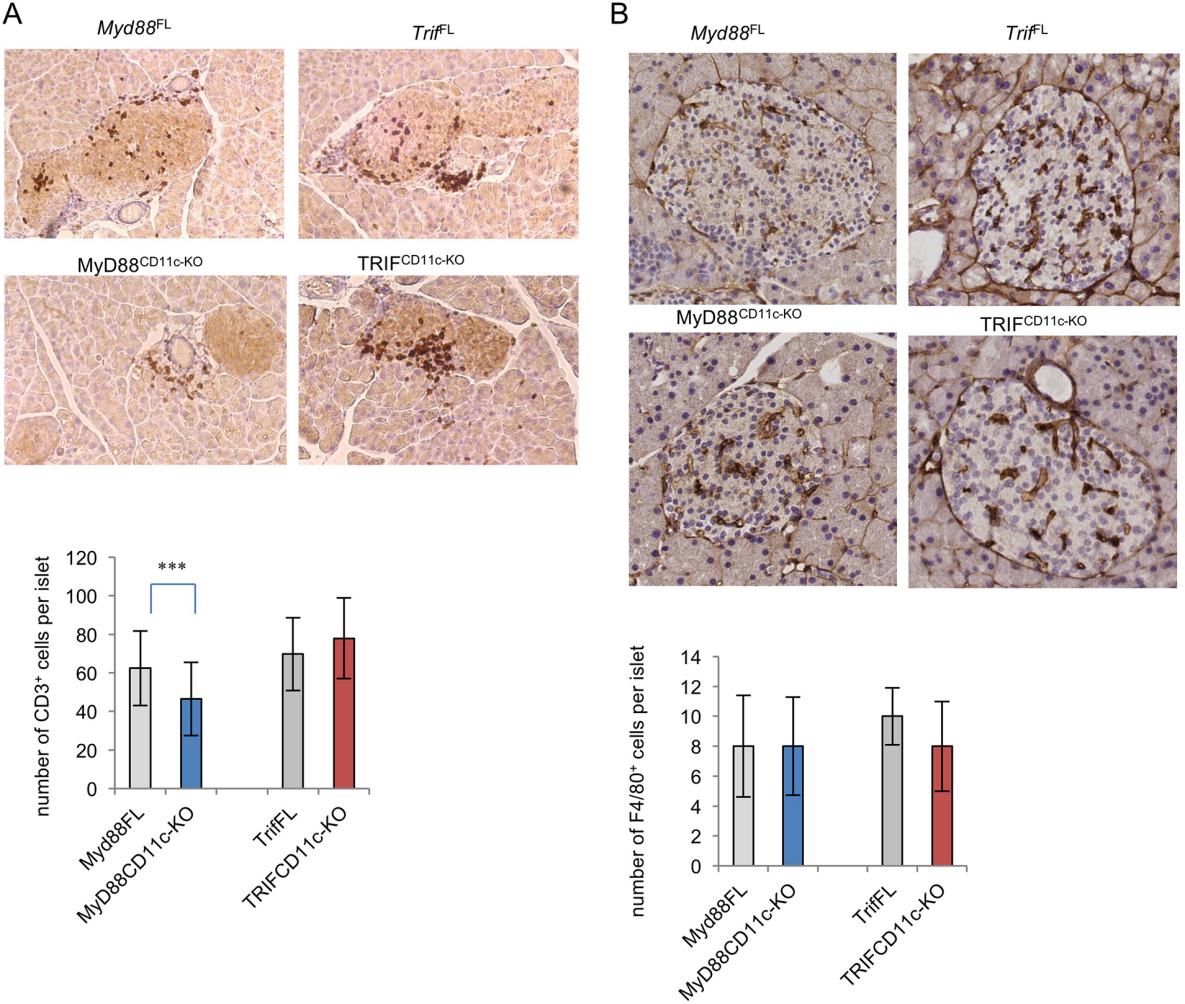
Immune cell infiltration in pancreatic islets of STZ-treated mice. Paraffin sections of pancreatic islets collected from mice one week after completion of the STZ treatment were stained for CD3 (A) or F4/80 (B). CD3^+^ or F4/80^+^ cells were counted on 30–50 islets per genotype of mice scored with insulitis. *Myd88*^FL^ (n = 4), MyD88^CD11c-KO^ (n = 3), *Trif*^FL^ (n = 5) and TRIF^CD11c-KO^ (n = 6).

We then compared the presence of regulatory T cells (Tregs) in the PLNs of STZ-treated MyD88^CD11c-KO^ and TRIF^CD11c-KO^ mice. As shown in Fig 2B, the percentages of Tregs in the PLNs of TRIF^CD11c-KO^ mice were considerably reduced compared to their littermate controls one week after completion of the STZ injections. MyD88^CD11c-KO^ mice showed a trend towards increased percentages of Tregs compared to their littermate controls, although this difference was not statistically significant (Fig 2B). Assessment of T cell infiltration in pancreatic islets of mice one week after the completion of STZ treatment revealed a trend towards slightly increased numbers of T cells in TRIF^CD11c-KO^ mice, while islets from MyD88^CD11c-KO^ mice contained less T cells compared to their respective littermate controls (Fig 3A). Immunostaining for F4/80 revealed similar numbers of macrophages in islets from MyD88^CD11c-KO^, TRIF^CD11c-KO^ and littermate control mice (Fig 3B). Considering that accumulation of diabetogenic T cells in pancreatic islets is a hallmark of the pathogenesis of T1D, these results indicate that myeloid MyD88 signaling promotes the development of STZ-induced diabetes by negatively regulating *Ido* expression and Treg induction in draining PLNs.

### MyD88^-/-^ and TRIF^-/-^ dendritic cells respond differently to apoptotic cell phagocytosis

TLR2-dependent sensing of dead β-cells by APCs has been suggested to provide a critical initial stimulus for the development of autoimmune diabetes [10]. We hypothesized that MyD88 or TRIF deficiency in myeloid cells may exert opposing effects on the development of STZ-induced diabetes by differentially regulating the responses of antigen presenting cells to apoptotic beta-cells. We therefore tested the responses of MyD88^-/-^ and TRIF^-/-^ DCs to apoptotic cells *in vitro*. For this purpose, apoptotic splenocytes were prepared by staurosporine treatment (see methods) and were ‘fed’ to wild type (WT), MyD88^-/-^ or TRIF^-/-^ bone marrow-derived dendritic cells (BMDCs). Phagocytosis assays showed that MyD88^-/-^ and TRIF^-/-^ cells could take up apoptotic splenocytes with the same efficiency as WT cells (S3 Fig). Similar results were obtained when using IgG-coated latex beads instead of primary apoptotic cells. Thus, MyD88 or TRIF deficiency did not affect the capacity of BMDCs to take up apoptotic cells.

**Fig 4:**
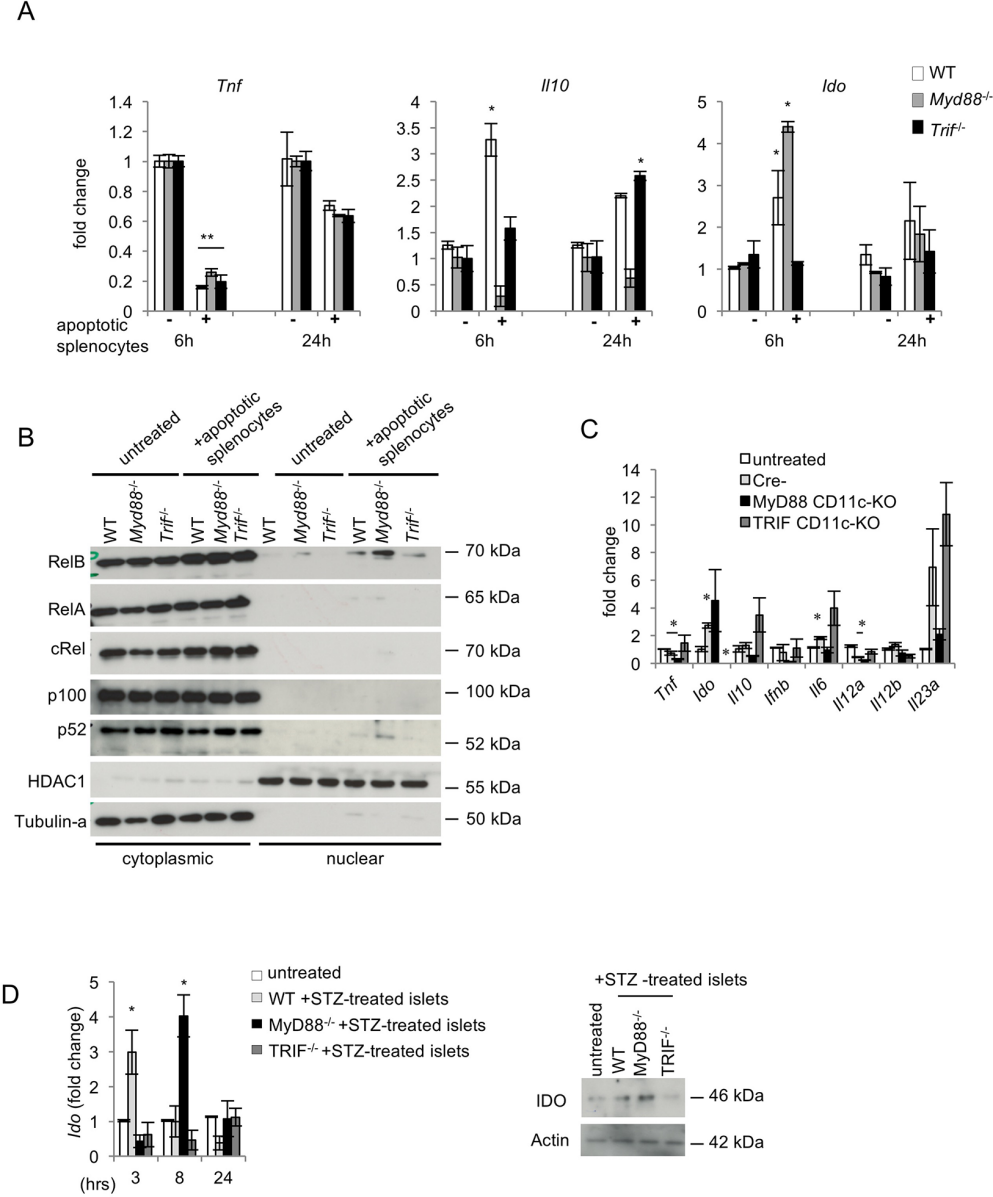
Differential response of MyD88- or TRIF-deficient DCs to apoptotic cell phagocytosis. (A) WT, *Myd88*^-/-^ and *Trif*^-/-^ BMDCs were stimulated with apoptotic splenocytes for 6 or 24 hours and the expression of *Tnf*, *Il10* and *Ido* mRNAs was measured by qRT-PCR. Data shown are representative of three independent experiments. (B) The expression of NF-κB proteins was assessed by immunoblotting with specific antibodies in cytoplasmic and nuclear extracts from BMDCs stimulated with apoptotic splenocytes for 4 hours. Tubulin and HDAC1 were used as loading controls. Blots are representative of three independent experiments. (C) The mRNA expression of the indicated genes was measured by qRT-PCR in peritoneal cells collected from MyD88^CD11c-KO^, TRIF^CD11c-KO^ and Cre negative littermate control mice (n = 4 per genotype) 18 hours after i.p. injection of 10^7^ apoptotic thymocytes or medium-only. Data are representative of two independent experiments. (D) The mRNA and protein expression of IDO was analyzed by qRT-PCR and immunoblotting respectively in WT, *Myd88*^-/-^ and *Trif*^-/-^ BMDCs that were stimulated with primary apoptotic islets (immunoblot was performed on protein extracts prepared from islets stimulated for 12 hours with STZ). Data are representative of two independent experiments.

Apoptotic cell clearance by phagocytes is considered an immunologically silent event [28–30]. Indeed, apoptotic splenocytes decreased MHCII and *Tnf* expression and suppressed LPS-induced pro-inflammatory cytokine production in BMDCs, but these responses were not altered by MyD88 or TRIF deficiency (Fig 4A and S3 Fig). Furthermore, apoptotic splenocytes induced upregulation of *Il10* expression in BMDCs, which was blunted in the absence of MyD88. *Ido* expression was moderately induced in BMDCs in response to stimulation with apoptotic splenocytes. Interestingly, consistent with the differential effect of MyD88 or TRIF deficiency on *Ido* expression in PLNs from STZ-treated mice, apoptotic cell-induced expression of *Ido* was increased in MyD88-deficient BMDCs and decreased in TRIF-deficient BMDCs (Fig 4A).

*Ido* expression is controlled by the non-canonical NF-κB pathway [31, 32] but also through STAT-1 signaling [33–35]. We therefore examined the activation of NF-κB and STAT-1 in BMDCs treated with apoptotic splenocytes. These experiments showed that apoptotic cell stimulation induced nuclear translocation of RelB and p52 but not of RelA in WT cells, which was enhanced in the absence of MyD88 but decreased in TRIF-deficient BMDCs (Fig 4B). These results suggest that MyD88 and TRIF exert opposing effects on *Ido* expression by differentially regulating NF-κB signaling in BMDCs in response to stimulation by apoptotic cells.

To verify these findings *in vivo* we examined the responses of mice injected with apoptotic cells. We found that intraperitoneal administration of apoptotic thymocytes *in vivo* induced a similar cytokine profile in resident peritoneal cells (Fig 4C). Interestingly, resident peritoneal cells from TRIF^CD11c-KO^ mice displayed increased expression of cytokines such as *l10*, *Il6 and Il23a* while MyD88^CD11c-KO^ mice showed reduced cytokine expression compared to the respective control animals in response to apoptotic thymocyte injection. Consistent with our *in vitro* findings, myeloid-cell specific TRIF deficiency almost completely abolished *Ido* upregulation in resident peritoneal cells after injection of apoptotic thymocytes, while myeloid cell specific MyD88 deficiency had an opposite effect resulting in increased expression of *Ido* compared to the control animals.

Finally, in order to test a type of apoptotic cells that is more relevant to the STZ model, we isolated primary pancreatic islets and stimulated them with STZ *in vitro*. WT, *Myd88*^-/-^ or *Trif*^-/-^ BMDCs were subsequently stimulated with the apoptotic β-cells and gene expression at different time points was examined. Importantly, stimulation with apoptotic islets induced *Ido* expression both at the transcriptional and protein level in WT and *Myd88*^-/-^ BMDCs but not in *Trif*^-/-^ cells (Fig 4D). Taken together, these results show that MyD88 and TRIF differentially regulate the expression of *Ido* in BMDCs exposed to apoptotic cells.

### Role of MyD88 and TRIF in myeloid cells in diabetes development in NOD mice

To study the role of myeloid-cell specific TLR signaling in the development of autoimmune diabetes in NOD mice, we backcrossed *Myd88*^FL^, *Trif*^FL^, *LysM-Cre* and *CD11c-Cre* into the NOD genetic background for at least 10 generations before intercrossing them to generate NOD.MyD88^LysM-KO^, NOD.MyD88^CD11c-KO^, NOD.TRIF^LysM-KO^ and NOD.TRIF^CD11c-KO^ mice. FACS analysis confirmed that the efficiency and specificity of Cre-mediated deletion of the MyD88 and TRIF floxed alleles in the NOD genetic background was similar compared with the original mouse lines generated in C57BL/6 genetic background.

**Fig 5:**
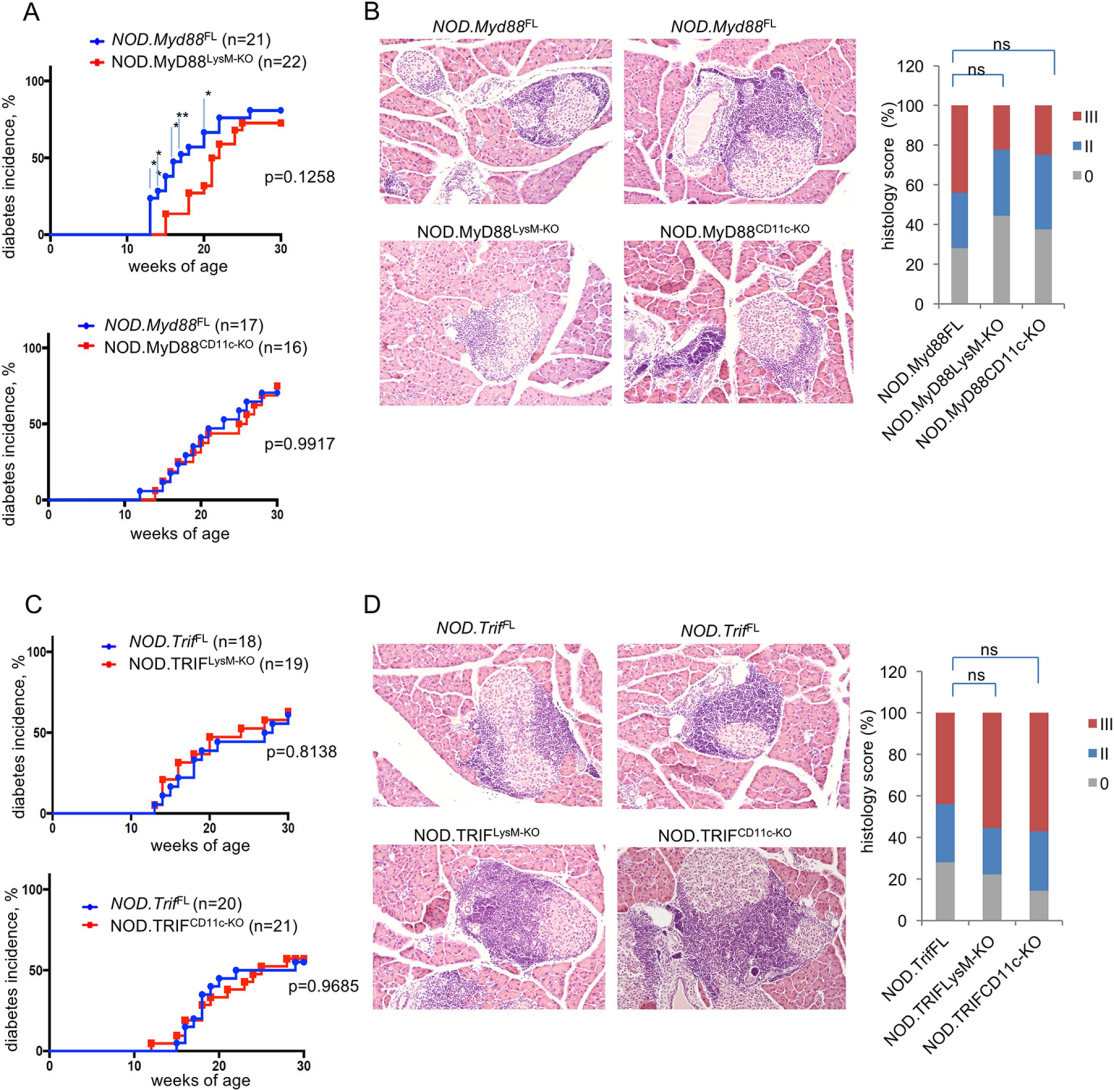
Development of autoimmune diabetes in NOD mice with myeloid- specific MyD88 or TRIF deficiency. (A and C) Graphs depicting the incidence of diabetes in mice with the indicated genotypes. (B and D) Representative images and quantification of H&E stained paraffin sections of pancreatic tissue from 10-week-old mice with the indicated genotypes. Graph depicts the percentage of mice with a given histology score per genotype: 0 or I, no islet infiltrates or only small peri-islet infiltrates; II, invasive insulitis (<50% of islet area); III, severe invasive insulitis (>50% of islet area). 20–30 islets per mouse were counted. NOD.MyD88^CD11c-KO^ (n = 8), NOD.MyD88^LysM-KO^ (n = 9), NOD.TRIF^CD11c-KO^ (n = 7) and NOD.TRIF^LysM-KO^ (n = 9), *NOD*.*Myd88*^FL^ (n = 13), and *NOD*.*Trif*^FL^ (n = 12).

Diabetes development in NOD mice occurs spontaneously at a rate of 60–80% in females and about 20% in males [2]. We therefore studied diabetes development in females in order to reduce the number of mice needed. NOD.MyD88^LysM-KO^, NOD.MyD88^CD11c-KO^, NOD.TRIF^LysM-KO^, NOD.TRIF^CD11c-KO^ mice and their littermate controls carrying MyD88 or TRIF floxed alleles but not the respective Cre trangenes were monitored for diabetes development by weekly blood glucose measurements starting from the age of 8–10 weeks. NOD.MyD88^LysM-KO^ mice showed considerably delayed development of diabetes until the age of 20 weeks, when only about 25% of the mice were diabetic compared to about 60% of the controls (Fig 5A). However, after 20 weeks the occurrence of diabetes in NOD.MyD88^LysM-KO^ mice increased and by the age of 30 weeks they showed similar diabetes incidence compared to their littermate controls. In contrast, NOD.MyD88^CD11c-KO^ mice developed diabetes with the same kinetics and overall incidence as their littermate controls (Fig 5A).

Diabetes development in NOD mice is preceded by immune cell infiltration into pancreatic islets. We therefore assessed whether MyD88 deficiency in myeloid cells affected islet infiltration. Histological analysis of pancreatic sections from 10 week-old mice revealed a trend towards reduced immune infiltration in islets of NOD.MyD88^LysM-KO^ mice compared to their littermate controls (Fig 5B), consistent with the delayed development of diabetes in these animals. Moreover, we found that also NOD.MyD88F^CD11c-KO^ mice showed a similar trend towards reduced immune cell infiltration in pancreatic islets at the age of 10 weeks, although the kinetics and overall diabetes incidence in these mice were not different than their littermate controls.

NOD.TRIF^LysM-KO^ and NOD.TRIF^CD11c-KO^ mice developed diabetes with similar kinetics and overall incidence compared to their respective littermate controls (Fig 5C), suggesting that TRIF signaling in myeloid cells does not play an important role in the development of diabetes in NOD mice. Consistent with these findings, NOD.TRIF^LysM-KO^ and NOD.TRIF^CD11c-KO^ mice showed similar immune cell infiltration in pancreatic islets at 10 weeks of age (Fig 5D). Taken together, these results showed that myeloid cell specific MyD88 signaling contributes to the development of diabetes in NOD mice by accelerating the onset of the disease.

## Discussion

Here we addressed the myeloid cell-specific role of MyD88- and TRIF-dependent TLR signaling in the pathogenesis of T1D in the NOD and STZ-induced mouse models of the disease. Interestingly, our results revealed that MyD88 signaling in myeloid cells plays an important pathogenic role for the development of T1D in the STZ model, but is less critical for T1D development in the NOD model where it seems to contribute by accelerating the onset but is not required for the overall incidence and development of the disease. These results are in contrast to earlier studies in mice with systemic MyD88 deficiency, which showed that MyD88 deficiency was protective in NOD mice but worsened STZ-induced pancreatic islet injury and T1D development, suggesting that MyD88 plays different roles in the NOD and STZ models [18], [20]. This discrepancy is likely due to the additional roles of MyD88 in regulating the microbiota but also the size of pancreatic islets and their response to injury, which complicates the interpretation of the results obtained using complete knockout animals.

The protective effect of myeloid-specific MyD88 deficiency in NOD mice was transient, manifesting with significant delay in the development of diabetes, but the mice eventually developed the disease. In contrast, myeloid specific MyD88 knockout not only delayed but also reduced the overall incidence of the disease in the STZ model. The discrepancy between the results in the two different models could be explained by the fact that NOD mice harbor several defects in central tolerance mechanisms which allow the persistence of autoreactive T-cells prior to any autoimmune inflammatory process. It is plausible that these genetic susceptibility factors can eventually by-pass the requirement for MyD88 in the myeloid compartment. Such parameters may not be present in the STZ model, which relies on the local autoinflammatory process rather than a genetically determined systemic immune deregulation. Therefore, it is likely that differences in disease incidence due to the absence of MyD88 in the NOD and STZ models are masked by other factors. Despite this difference, our results provide *in vivo* experimental evidence that MyD88 signaling in myeloid cells plays a critical pathogenic role in the development of autoimmune diabetes. Using cell depletion studies, Diana et al recently showed that a crosstalk between different innate immune cell types, such as neutrophils, plasmacytoid DCs and B1a cells, contribute to the initiation of autoimmune diabetes in NOD mice, and that MyD88 plays a critical role in the activation of pDCs [36]. These results are consistent with our findings showing that ablation of MyD88 signaling specifically in myeloid cells delayed the onset of T1D in NOD mice, and suggest that MyD88 likely acts also on other cell types to regulate autoimmunity in this model.

Previous studies in mice with systemic TRIF deficiency suggested that TRIF signaling mediates a microbiota-dependent protective effect in the development of T1D in NOD mice [19]. TRIF deficient mice were also shown to have increased β-cell mass and suffer from hyperglycemia associated with β-cell dysfunction, which makes difficult the study of the possible role of TRIF in autoimmune processes leading to diabetes using mice lacking TRIF systemically [21]. Our results showed that myeloid cell-specific TRIF deficiency resulted in a trend towards accelerated development of diabetes in the STZ model. Although these differences did not reach statistical significance, these findings indicate that TRIF signaling exerts a protective effect and delays the development of islet autoimmunity.

Our results suggest that the differential role of MyD88 and TRIF in the pathogenesis of autoimmune diabetes likely depends, at least in part, on the opposite effects of MyD88 and TRIF deficiency on the expression of IDO. We found that the expression of *Ido* in the PLNs of STZ- treated TRIF^CD11c-KO^ mice was significantly reduced compared to similarly treated littermate control mice. In contrast, *Ido* expression was increased in the PLNs of STZ- treated MyD88^CD11c-KO^ mice compared to their *Myd88*^FL^ littermates. Interestingly, the frequency of regulatory T cells was decreased in the PLNs of STZ- treated TRIF^CD11c-KO^ mice but was increased in PLNs of STZ- treated MyD88^CD11c-KO^ mice. These results could be linked to each other since the immunosuppressive activity of IDO drives the expansion of regulatory T cells [37].

IDO is rapidly expressed by DCs in response to various signals [38–40] but the role of MyD88 and TRIF in IDO induction is not clear. In synovial fibroblasts, poly (I:C), a ligand of TLR3 that induces TRIF-dependent signaling, appeared to induce increased expression of IDO compared to LPS, while MyD88 blockade was found to upregulate IDO expression [41]. Another study showed that IDO protein could not be detected in TRIF-deficient DCs pulsed with a fungus [42]. Altogether these studies suggested that *Ido* expression is dependent on TRIF signaling. In agreement to these studies and in accordance to our *in vivo* data, we found that TRIF-deficient DCs showed markedly impaired *Ido* expression in response to apoptotic cell phagocytosis *in vitro*. Moreover, MyD88 deficiency had the opposite effect resulting in considerably increased *Ido* expression in DCs stimulated with apoptotic cells. Apoptotic cell clearance is believed to play crucial role in autoimmune disease development and, importantly, IDO has been implicated in the regulation of tolerance to apoptotic cells thereby inhibiting systemic autoimmune disease development [43]. Specifically regarding T1D, it has been shown that IDO mediates protection from experimental autoimmune diabetes [44] and that impaired IDO expression and thus tryptophane catabolism underlies defective tolerance in NOD mice [45]. Also, it was recently reported that IDO-expressing fibroblasts can reverse diabetes in NOD mice [46]. Collectively, these studies show that IDO is a potent immunomodulatory molecule with an important role in the mechanisms determining the pathogenesis of autoimmunity, and suggest that the differential effect of MyD88 and TRIF on *Ido* expression in DCs exposed to apoptotic cells explains at least in part the differential functions of MyD88 and TRIF in diabetes development.

## Supporting information

S1 Fig Assessment of Cre-mediated deletion of MyD88 in myeloid cells. (A) Representative FACS plots of the analysis of resident peritoneal cells or splenocytes from 6–8 MyD88^CD11c-KO^ and MyD88^LysM-KO^ mice and their *Myd88*^FL^ littermates. The y axis on the plots indicates the respective live, gated populations and the x axis the GFP signal. (B) Table depicts percentages (%) of GFP^+^ or mCherry^+^ cells within the CD11b^+^, CD11c^+^ and F4/80^+^ resident peritoneal cells, or CD3^+^, CD19^+^ and CD11b^+^Gr1^+^ splenocytes, as determined by FACS analysis. (C) Western blot analysis of lysates from FACS sorted (as indicated) resident peritoneal cells from *Myd88*^FL^, MyD88^LysM-KO^ or MyD88^CD11c-KO^ mice. (D) Quantitative PCR analysis of FACS sorted and stimulated for 8 hours with 10 ng/ml LPS resident peritoneal cells from *Myd88*^FL^ (n = 3), MyD88^LysM-KO^(n = 2) or *Myd88*^-/-^ (n = 2) mice.
(TIFF)

S2 Fig Assessment of Cre-mediated deletion of TRIF in myeloid cells. (A) Representative FACS plots of the analysis of resident peritoneal cells or splenocytes from 6–8 TRIF^CD11c-KO^ and TRIF^LysM-KO^ mice and their control littermates *Trif*^FL^ mice. The y axis on the plots indicates the respective live, gated populations and the x axis the mCherry signal. (B) Table depicts percentages (%) of mCherry^+^ cells within the CD11b^+^, CD11c^+^ and F4/80^+^ resident peritoneal cells, or CD3^+^, CD19^+^ and CD11b^+^Gr1^+^ splenocytes, as determined by FACS analysis.
(TIFF)

S3 Fig Role of Myd88 and TRIF in the regulation of DC responses to apoptotic cells. (A) Phagocytosis of apoptotic cells. FACS analysis of WT, MyD88^-/-^ and TRIF^-/-^ BMDCs 3 and 8 hours after stimulation with primary apoptotic splenocytes (1:1) labeled with CA-FITC. (B) Apoptotic cell phagocytosis induces downregulation of MHCII in DCs. FACS analysis of WT, MyD88^-/-^ and TRIF^-/-^ BMDCs 12 hours after stimulation with primary apoptotic splenocytes (1:1), TNF, or combination. Bars indicate percentages of CD11c^+^ cells expressing MHCII before and after apoptotic cell administration. (C) Apoptotic cell phagocytosis induces downregulation of LPS-induced inflammatory cytokines in DCs. WT, MyD88^-/-^ and TRIF^-/-^ BMDCs were analyzed 6 and 24 hours after stimulation with primary apoptotic splenocytes (1:1), LPS, or combination of both. Gene expression was determined by qPCR analysis. In all experiments WT, *Myd88*^-/-^ and *Trif*^-/-^ primary cells were generated from 2 mice per genotype and used in triplicates (n = 6). All Experiments are representative of at least 4 repetitions.
(TIFF)
