# Supplementary figures and images for: Differential role of MyD88 and TRIF signaling in myeloid cells in the pathogenesis of autoimmune diabetes
Source: PLoS One. 2018 Mar 9;13(3):e0194048. doi: 10.1371/journal.pone.0194048 (PMC5844544; doi:10.1371/journal.pone.0194048)

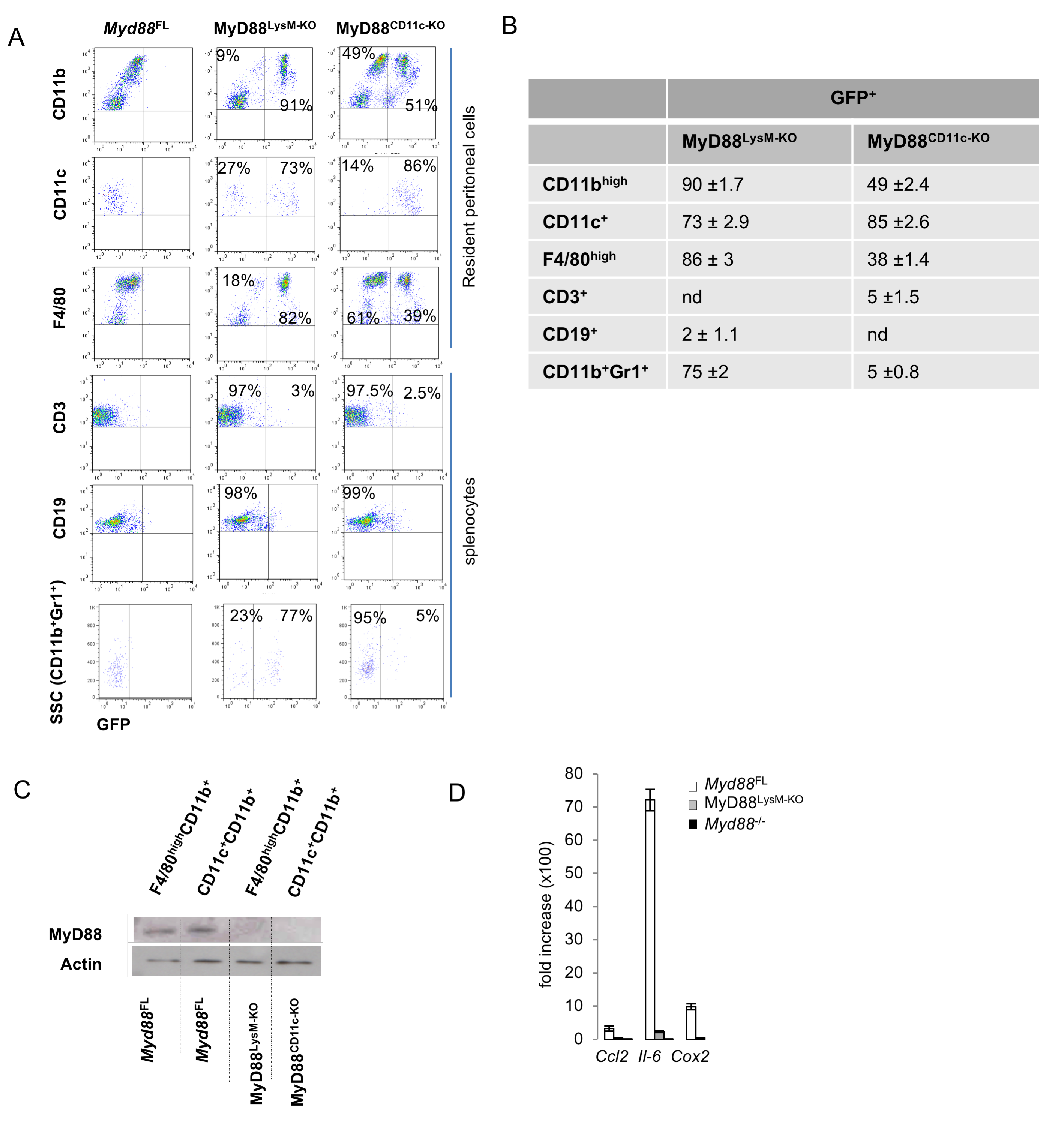

Supplement: S1 Fig — (A) Representative FACS plots of the analysis of resident peritoneal cells or splenocytes from 6–8 MyD88CD11c-KO and MyD88LysM-KO mice and their Myd88FL littermates. The y axis on the plots indicates the respective live, gated populations and the x axis the GFP signal. (B) Table depicts percentages (%) of GFP+ or mCherry+ cells within the CD11b+, CD11c+ and F4/80+ resident peritoneal cells, or CD3+, CD19+ and CD11b+Gr1+ splenocytes, as determined by FACS analysis. (C) Western blot analysis of lysates from FACS sorted (as indicated) resident peritoneal cells from Myd88FL, MyD88LysM-KO or MyD88CD11c-KO mice. (D) Quantitative PCR analysis of FACS sorted and stimulated for 8 hours with 10 ng/ml LPS resident peritoneal cells from Myd88FL (n = 3), MyD88LysM-KO(n = 2) or Myd88-/- (n = 2) mice. (TIFF) [file pone.0194048.s001.tiff]

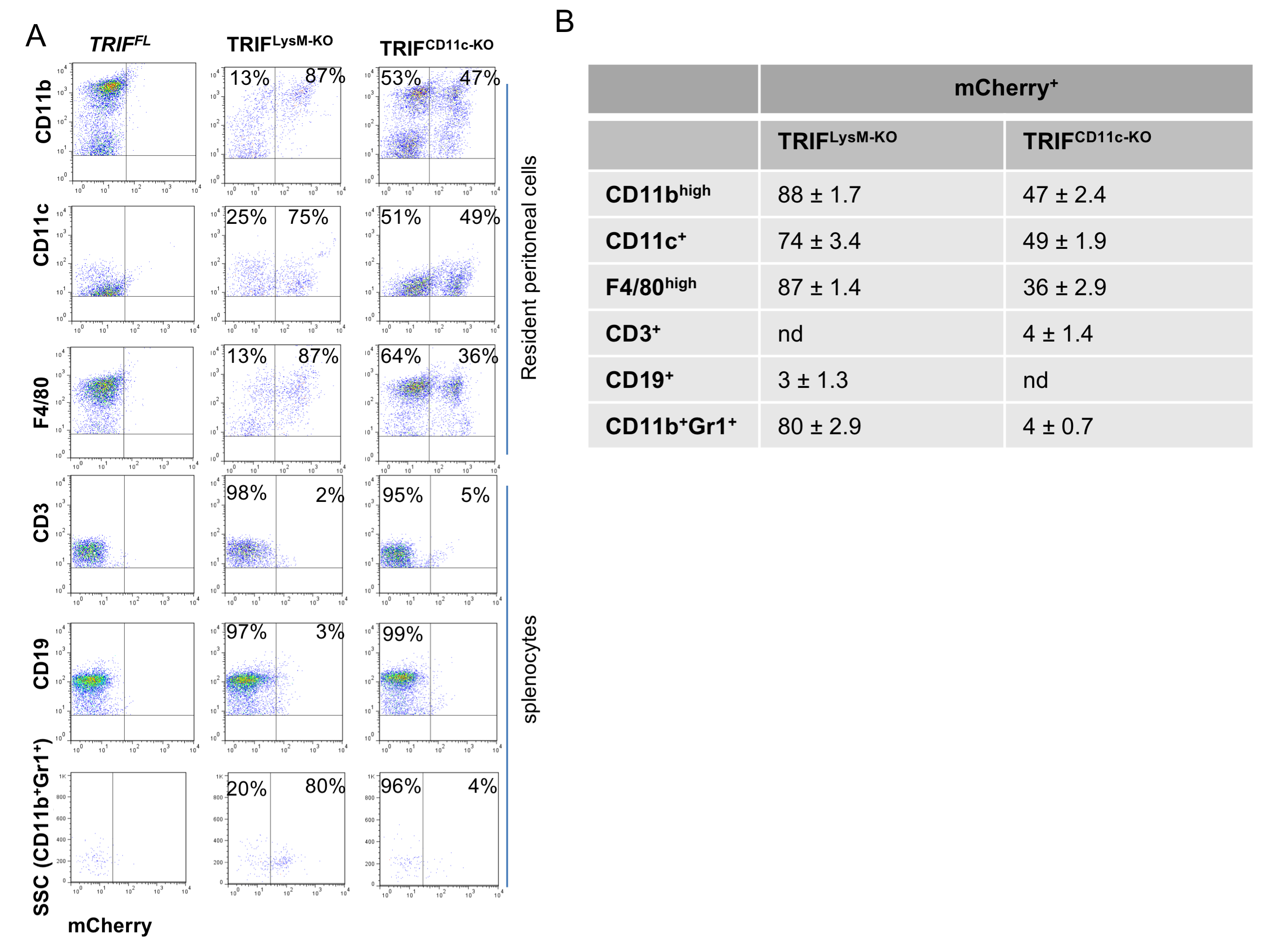

Supplement: S2 Fig — (A) Representative FACS plots of the analysis of resident peritoneal cells or splenocytes from 6–8 TRIFCD11c-KO and TRIFLysM-KO mice and their control littermates TrifFL mice. The y axis on the plots indicates the respective live, gated populations and the x axis the mCherry signal. (B) Table depicts percentages (%) of mCherry+ cells within the CD11b+, CD11c+ and F4/80+ resident peritoneal cells, or CD3+, CD19+ and CD11b+Gr1+ splenocytes, as determined by FACS analysis. (TIFF) [file pone.0194048.s002.tiff]

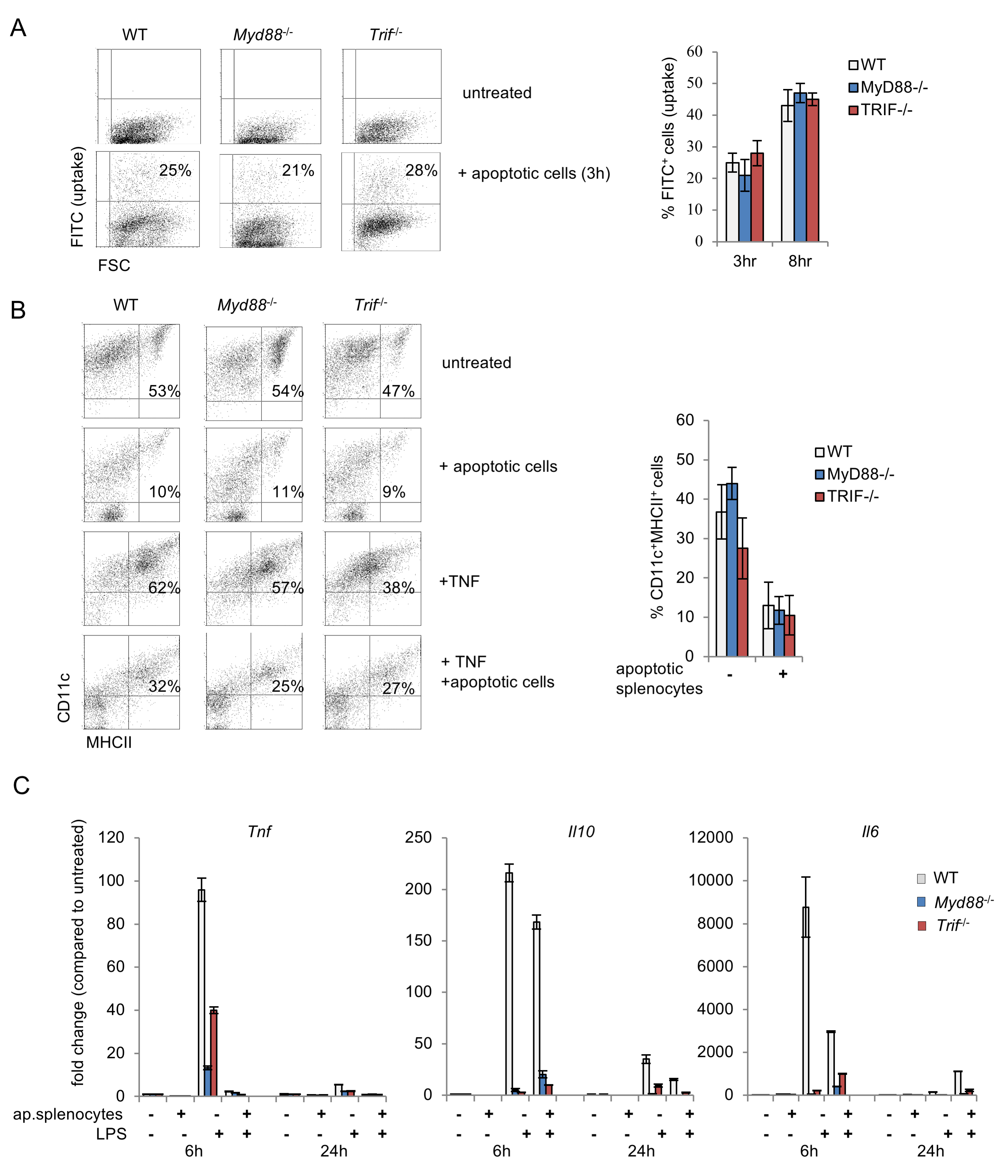

Supplement: S3 Fig — (A) Phagocytosis of apoptotic cells. FACS analysis of WT, MyD88-/- and TRIF-/- BMDCs 3 and 8 hours after stimulation with primary apoptotic splenocytes (1:1) labeled with CA-FITC. (B) Apoptotic cell phagocytosis induces downregulation of MHCII in DCs. FACS analysis of WT, MyD88-/- and TRIF-/- BMDCs 12 hours after stimulation with primary apoptotic splenocytes (1:1), TNF, or combination. Bars indicate percentages of CD11c+ cells expressing MHCII before and after apoptotic cell administration. (C) Apoptotic cell phagocytosis induces downregulation of LPS-induced inflammatory cytokines in DCs. WT, MyD88-/- and TRIF-/- BMDCs were analyzed 6 and 24 hours after stimulation with primary apoptotic splenocytes (1:1), LPS, or combination of both. Gene expression was determined by qPCR analysis. In all experiments WT, Myd88-/- and Trif-/- primary cells were generated from 2 mice per genotype and used in triplicates (n = 6). All Experiments are representative of at least 4 repetitions. (TIFF) [file pone.0194048.s003.tiff]
